# Supplementary material for: Resolving the full spectrum of human genome variation using Linked-Reads
Source: Genome Res. 2019 Apr;29(4):635–45. doi: 10.1101/gr.234443.118 (PMC6442396; doi:10.1101/gr.234443.118)
Supplement: Supplemental Material [file supp_gr.234443.118_Supplemental_longranger-2.2.zip › longranger-2.2.tar.bz2!/lr2.2-public/lib/stan/cmdstan/stan/doc/api/html/matrix_2promote__scalar__type_8hpp_source.html]

Stan: src/stan/math/matrix/promote\_scalar\_type.hpp Source File


|  |  |
| --- | --- |
|  | Stan  2.5.0  probability, sampling & optimization |


- Main Page
- Related Pages
- Namespaces
- Classes
- Files

- File List
- File Members

All Classes Namespaces Files Functions Variables Typedefs Enumerations Enumerator Friends Macros Pages

- src
- stan
- math
- matrix

promote\_scalar\_type.hpp

Go to the documentation of this file.

1 #ifndef STAN\_\_MATH\_\_MATRIX\_\_PROMOTE\_SCALAR\_TYPE\_HPP

2 #define STAN\_\_MATH\_\_MATRIX\_\_PROMOTE\_SCALAR\_TYPE\_HPP

3

4 #include <vector>

5 #include <stan/math/functions/promote\_scalar\_type.hpp>

6 #include <stan/math/matrix/Eigen.hpp>

7

8 namespace stan {

9

10  namespace math {

11

22  template <typename T, typename S>

23  struct promote\_scalar\_type<T, Eigen::Matrix<S,Eigen::Dynamic,Eigen::Dynamic> > {

24

28  typedef Eigen::Matrix<typename promote\_scalar\_type<T,S>::type,

29  Eigen::Dynamic, Eigen::Dynamic>

30  type;

31

32  };

33

34

43  template <typename T, typename S>

44  struct promote\_scalar\_type<T, Eigen::Matrix<S,Eigen::Dynamic,1> > {

45

49  typedef Eigen::Matrix<typename promote\_scalar\_type<T,S>::type,

50  Eigen::Dynamic, 1>

51  type;

52

53  };

54

55

64  template <typename T, typename S>

65  struct promote\_scalar\_type<T, Eigen::Matrix<S,1,Eigen::Dynamic> > {

66

70  typedef Eigen::Matrix<typename promote\_scalar\_type<T,S>::type,

71  1, Eigen::Dynamic>

72  type;

73

74  };

75

76

77  }

78

79 }

80

81 #endif

stan::math::promote\_scalar\_type< T, Eigen::Matrix< S, 1, Eigen::Dynamic > >::type

Eigen::Matrix< typename promote\_scalar\_type< T, S >::type, 1, Eigen::Dynamic > type

The promoted type.

**Definition:** promote\_scalar\_type.hpp:72

promote\_scalar\_type.hpp

stan::math::promote\_scalar\_type

Template metaprogram to calculate a type for converting a convertible type.

**Definition:** promote\_scalar\_type.hpp:19

stan::math::promote\_scalar\_type< T, Eigen::Matrix< S, Eigen::Dynamic, Eigen::Dynamic > >::type

Eigen::Matrix< typename promote\_scalar\_type< T, S >::type, Eigen::Dynamic, Eigen::Dynamic > type

The promoted type.

**Definition:** promote\_scalar\_type.hpp:30

Eigen.hpp

stan::math::promote\_scalar\_type< T, Eigen::Matrix< S, Eigen::Dynamic, 1 > >::type

Eigen::Matrix< typename promote\_scalar\_type< T, S >::type, Eigen::Dynamic, 1 > type

The promoted type.

**Definition:** promote\_scalar\_type.hpp:51


---

[ Stan Home Page ]

*© 2011–2014,
Stan Development Team.*
